# Neuroprotective Effects against Glutamate-Induced HT-22 Hippocampal Cell Damage and *Caenorhabditis elegans* Lifespan/Healthspan Enhancing Activity of *Auricularia polytricha* Mushroom Extracts

**DOI:** 10.3390/ph14101001

**Published:** 2021-09-29

**Authors:** Chanin Sillapachaiyaporn, Panthakarn Rangsinth, Sunita Nilkhet, Alison T. Ung, Siriporn Chuchawankul, Tewin Tencomnao

**Affiliations:** 1 Program in Clinical Biochemistry and Molecular Medicine, Department of Clinical Chemistry, Faculty of Allied Health Sciences, Chulalongkorn University, Bangkok 10330, Thailand; 6271002637@student.chula.ac.th (C.S.); 6273006537@student.chula.ac.th (S.N.); 2 Department of Transfusion Medicine and Clinical Microbiology, Faculty of Allied Health Sciences, Chulalongkorn University, Bangkok 10330, Thailand; panthakarn.r@chula.ac.th; 3 School of Mathematical and Physical Sciences, Faculty of Science, The University of Technology Sydney, Sydney, NSW 2007, Australia; Alison.Ung@uts.edu.au; 4 Immunomodulation of Natural Products Research Group, Faculty of Allied Health Sciences, Chulalongkorn University, Bangkok 10330, Thailand; 5 Department of Clinical Chemistry, Faculty of Allied Health Sciences, Chulalongkorn University, Bangkok 10330, Thailand; 6 Natural Products for Neuroprotection and Anti-Ageing (Neur-Age Natura) Research Unit, Faculty of Allied Health Sciences, Chulalongkorn University, Bangkok 10330, Thailand

**Keywords:** *Auricularia polytricha*, glutamate toxicity, antioxidant activity, neuroprotective effect, *Caenorhabditis elegans*, lifespan extension, healthspan improvement, linoleic acid

## Abstract

Oxidative stress is associated with several diseases, particularly neurodegenerative diseases, commonly found in the elderly. The attenuation of oxidative status is one of the alternatives for neuroprotection and anti-aging. *Auricularia polytricha* (AP), an edible mushroom, contains many therapeutic properties, including antioxidant properties. Herein, we report the effects of AP extracts on antioxidant, neuroprotective, and anti-aging activities. The neuroprotective effect of AP extracts against glutamate-induced HT-22 neuronal damage was determined by evaluating the cytotoxicity, intracellular reactive oxygen species (ROS) accumulation, and expression of antioxidant enzyme genes. Lifespan and healthspan assays were performed to examine the effects of AP extracts from *Caenorhabditis elegans*. We found that ethanolic extract (APE) attenuated glutamate-induced HT-22 cytotoxicity and increased the expression of antioxidant enzyme genes. Moreover, APE promoted in the longevity and health of the *C. elegans*. Chemical analysis of the extracts revealed that APE contains the highest quantity of flavonoids and a reasonable percentage of phenols. The lipophilic compounds in APE were identified by gas chromatography/mass spectrometry (GC/MS), revealing that APE mainly contains linoleic acid. Interestingly, linoleic acid suppressed neuronal toxicity and ROS accumulation from glutamate induction. These results indicate that AP could be an exciting natural source that may potentially serves as neuroprotective and anti-aging agents.

## 1. Introduction

Mushrooms are rich sources of nutritional compounds that possess medicinal properties [1,2]. *Auricularia* spp, also known as “wood ear” edible mushrooms, are commonly used in Asian cooking and traditional medicine [3]. Auricularia mushrooms have been reported to have antiviral [4], antitumoral [5,6], and immune-enhancing activities [7]. Many studies have shown that *Auricularia polytricha* (AP) contained antioxidant compounds [8,9,10,11]. Aqueous extracts of AP have attenuated paracetamol-induced hepatotoxicity and hepatic lipid accumulation in rat models due to their antioxidative effects [12,13]. Furthermore, AP was known to provide therapeutic effects on neurodegenerative disease (NDD) progression by inhibiting *beta*-secretase (BACE-1) [14]. BACE-1 is an important enzyme involved in amyloid synthesis and the leading cause of Alzheimer’s disease, the common cause of NDD.

NDDs are caused by several factors, such as genetics, environments, inflammation, and the accumulation of oxidative stress [15]. Moreover, the imbalance of the neurotransmitter system is another leading cause of NDDs [16]. Glutamate is the essential excitatory neurotransmitter that regulates synaptogenesis. However, glutamate has been recognized to cause excitotoxicity at elevated concentrations, leading to neuronal cell death and synaptic loss, as well as neurodegeneration [17]. A high level of glutamate extracellular concentration could induce oxidative stress-mediated apoptosis [16]. Thus, inhibition of oxidative stress resulting from glutamate excitotoxicity may protect NDDs.

Based on the reported antioxidant activity of the AP mushroom, we hypothesize that the extracts of AP might have therapeutic effects on NDDs. This study aimed to investigate the neuroprotection and lifespan extension potential of AP. The neuroprotection aspect of the study involved evaluating the antioxidant and free radical scavenging activities of AP extracts. The neuroprotective potential of AP extracts was evaluated by the ability of the extracts to protect against HT-22 mouse hippocampal cell death resulting from glutamate-induced damage, intracellular reactive oxygen species (ROS) accumulation, and expression of antioxidant enzyme genes. At the same time, the lifespan and healthspan potentials of AP extracts were examined by performing assays on *Caenorhabditis elegans* longevity and health. Lipophilic compounds responsible for the activities observed in AP extracts were also analyzed by gas chromatography/mass spectrometry (GC/MS).

## 2. Results

### 2.1. Total Phenolic and Flavonoid Contents Present in AP Crude Extracts

**Figure 1:**
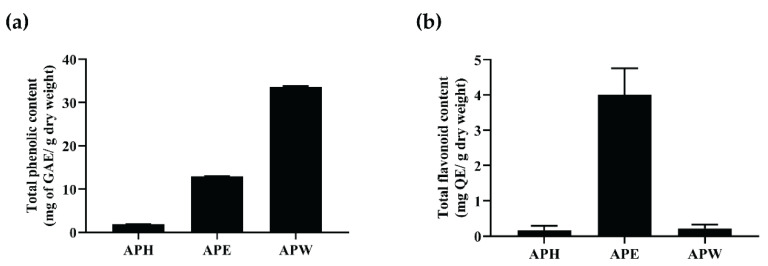
Total (**a**) phenolic and (**b**) flavonoid contents exist in AP crude extracts. All data are shown as the mean ± SEM of triplicate values.

AP was extracted by sequential maceration using solvents in increasing polarity. Three crude extracts were obtained: hexane (APH), ethanol (APE), and water (APW) crude extracts [4]. The flavonoid content in the three extracts was analyzed using aluminum chloride methods, while the water-soluble phenolic content was analyzed using the Folin-Ciocalteu method. The Folin-Ciocalteu method was used to analyze both flavonoids and other non-flavonoid compounds that contain phenolic groups. The results from both methods allowed the deduction of the content of water-soluble phenolic compounds that are non-flavonoids. The phenolic content analysis indicated that APW (33.66 ± 0.14 mg GAE/g dry weight) contained the highest level of phenolic compounds, followed by APE (12.95 ± 0.12 mg GAE/g dry weight) and APH (1.90 ± 0.03 mg GAE/g dry weight), respectively (Figure 1a). APE (4.01 ± 0.75 mg QE/g dry weight) exhibited the highest quantity of flavonoid content. In contrast, the flavonoid content was almost undetectable in APH and APW (Figure 1b). The results from the two experiments indicate that the water-soluble phenolic content in APW is mainly due to the non-flavonoid phenolic compounds.

### 2.2. Free Radical Scavenging Activity of AP Crude Extracts

The antioxidant properties of AP crude extracts were studied using ABTS and DPPH assays. Both assays relied on the capacity of the extracts to scavenge the free radicals and neutralize the reaction. The antioxidant capacity of AP extracts was expressed in mg of vitamin C equivalent antioxidant capacity (VCEAC)/g dry weight. As shown in Table 1, APH, APE, and APW had antioxidant capacity values of 0.92 ± 0.12 mg VCEAC/g dry weight, 1.57 ± 0.04 mg VCEAC/g dry weight, and 2.47 ± 0.06 mg VCEAC/g dry weight in ABTS assay, respectively. Statistical analysis indicated that APW had significantly higher ABTS scavenging activity than APH. In addition, APW showed the highest DPPH scavenging activity (1.88 ± 0.25 mg VCEAC/g dry weight), followed by APE (1.27 ± 0.04 mg VCEAC/g dry weight) and APH (0.99 ± 0.08 mg VCEAC/g dry weight).

The half-maximal effective concentration (EC_50_) values of APE and APW in the ABTS assay were 3.11 ± 0.05 mg/mL and 1.06 ± 0.04 mg/mL, respectively. For the DPPH assay, the EC_50_ values of APE and APW were 12.61 ± 3.47 mg/mL and 3.62 ± 0.35 mg/mL, respectively. However, at up to 8 mg/mL, APH showed no activity in both assays, and an accurate EC_50_ value was not obtainable. In comparison, APW was the most potent extract among the three AP extracts.

### 2.3. Cytotoxicity of AP Crude Extracts on HT-22 Mouse Hippocampal Cells

**Figure 2:**
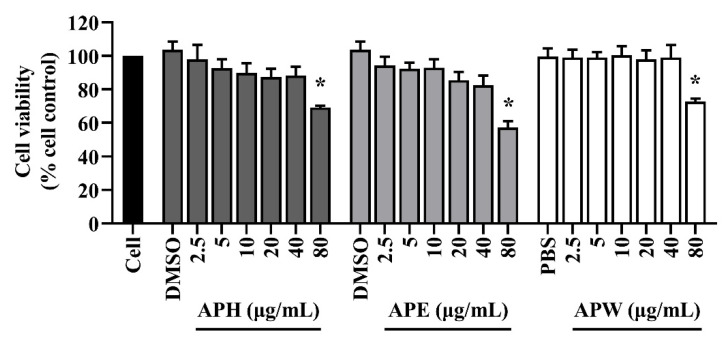
Cytotoxicity against HT-22 hippocampal cells of AP crude extracts: APH, APE, and APW for 24 h. DMSO (0.1%, *v*/*v*) and PBS (0.1%, *v*/*v*) were used as the vehicle control. All data are shown as the mean ± SEM of triplicate values. Statistical significance was analyzed by one-way ANOVA, Dunnett test. * *p* < 0.05 versus cell control.

The cytotoxicity of AP crude extracts on the HT-22 cells was determined by measuring the mitochondrial enzyme activity of live cells using colorimetric MTT assay. At 24 h of treatments, all extracts showed significant toxicity at an 80 µg/mL concentration (Figure 2). Therefore, any concentration lower than or equal to 40 µg/mL could be used as a safe concentration in the subsequent experiments.

### 2.4. Glutamate-Induced HT-22 Neurotoxicity and Neuroprotective Effects of AP Crude Extracts

**Figure 3:**
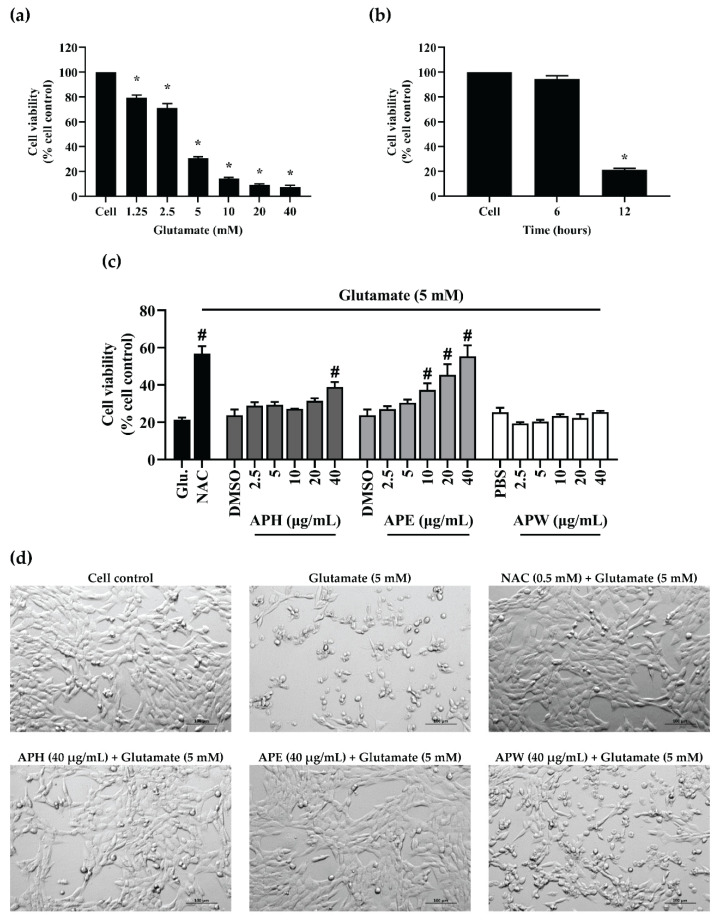
Glutamate-induced HT-22 hippocampal cell toxicity results. (**a**) Treatment of glutamate at various concentrations on HT-22 cells for 24 h. (**b**) Treatment of glutamate (5mM) on HT-22 cells for 6 and 12 h. (**c**) The effects of AP crude extracts: APH, APE, and APW on glutamate-induced HT-22 cells for 12 h. *N*-acetyl cysteine (NAC, 0.5 mM) was used as the positive control. DMSO (0.1%, *v*/*v*) and PBS (0.1%, *v*/*v*) were used as the vehicle control. All data are shown as the mean ± SEM of triplicate values. Statistical significance was analyzed by one-way ANOVA, Dunnett test. * *p* < 0.05 versus the cell control. # *p* < 0.05 versus the glutamate (Glu.) control. (**d**) Morphological changes of glutamate-induced HT-22 hippocampal cells after co-treatment with NAC (0.5 mM), APH (40 µg/mL), APE (40 µg/mL), or APW (40 µg/mL) for 12 h. The cells were observed under differential interference contrast (DIC) microscope at 5× magnification. The scale bar indicates 100 µm.

To examine HT-22 neurotoxicity induced by glutamate excitotoxicity, the level of glutamate that suppressed HT-22 cell viability was determined. At 1.25 mM to 40 mM of glutamate, HT-22 cell viability was significantly decreased in a dose-dependent manner comparing to the cell control. However, at an elevated concentration of 5 mM, glutamate caused approximately 70% neuronal toxicity (Figure 3a). This concentration was further used to induce HT-22 neuronal toxicity in this study.

The time dependence of glutamate treatment against HT-22 cells was also investigated. The results showed that at the concentration of 5 mM, glutamate efficiently killed HT-22 cells at 12 h of treatment compared to the untreated cell control. In contrast, at 6 h of treatment, the cell viability remained greater than 90% (Figure 3b). The contact time of glutamate on HT-22 cells was minimized by incubating the cells for 12 h. This was further utilized to induce glutamate excitotoxicity on HT-22 hippocampal cells.

At 40 µg/mL, APH significantly reduced glutamate toxicity by increasing the HT-22 cell viability compared to the glutamate control. In comparison, APE at 10, 20, and 40 µg/mL showed significant protection of HT-22 cells against glutamate-induced cell death in a dose-dependent manner. Interestingly, 40 µg/mL of APE exhibited the highest neuroprotective effect on glutamate-induced hippocampal cell toxicity. This effect is in line with *N*-acetyl cysteine (NAC), a well-known antioxidant compound. In contrast, APW (2.5–40 µg/mL) did not rescue HT-22 neurotoxicity from glutamate induction (Figure 3c). The morphological appearances of HT-22 cells treated with glutamate and AP extracts are shown in Figure 3d.

### 2.5. Intracellular Reactive Oxygen Species (ROS) Inhibitory Effect of AP Crude Extracts on Glutamate-Induced HT-22 Cell Toxicity

**Figure 4:**
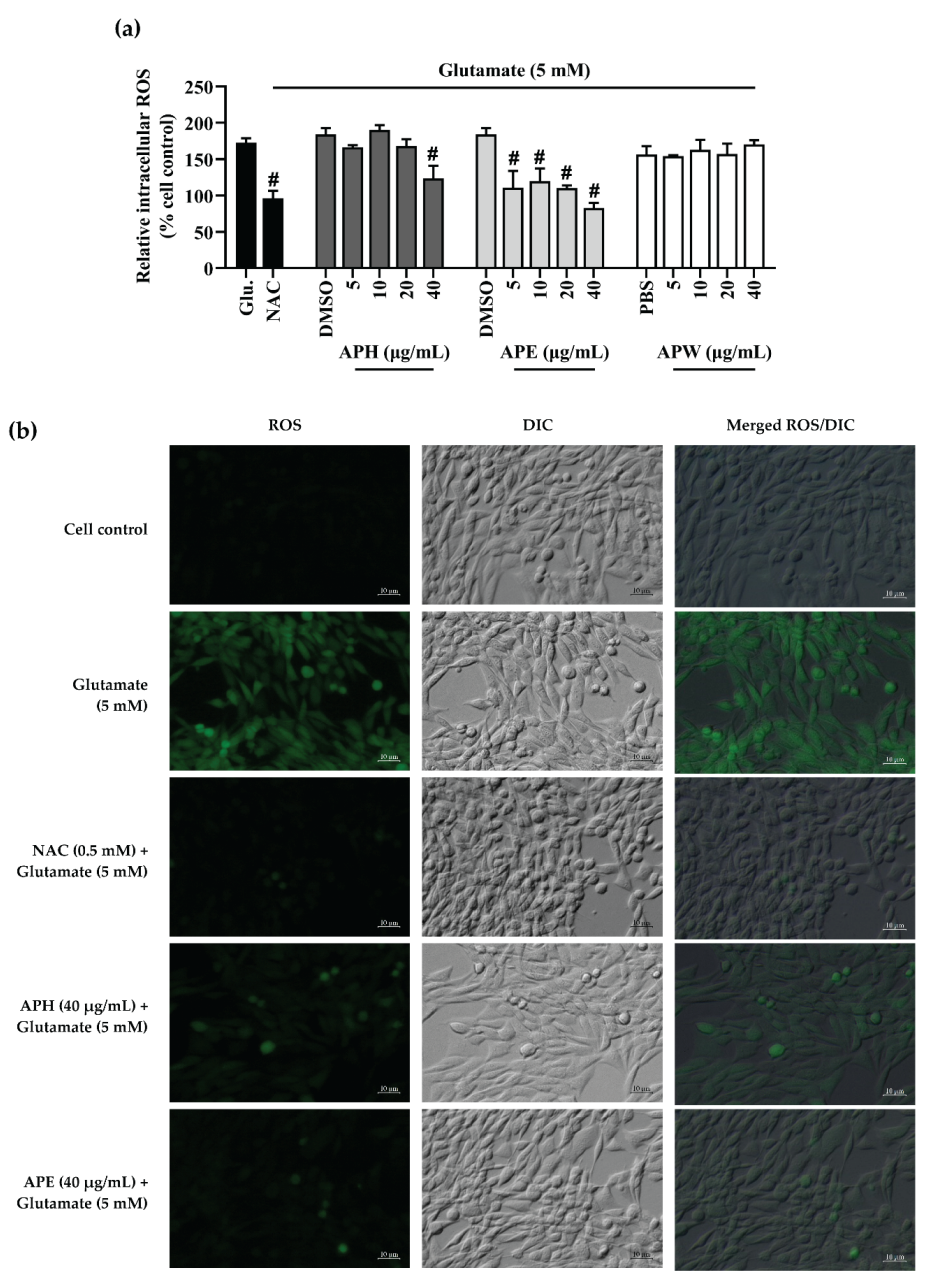
Intracellular reactive oxygen species (ROS) inhibitory effect of AP extracts co-treatment with 5 mM glutamate on HT-22 cells for 12 h. (**a**) The relative intracellular ROS of treatments compared to the cell control. *N*-acetyl cysteine (NAC, 0.5 mM) was used as the positive control. DMSO (0.1%, *v*/*v*) and PBS (0.1%, *v*/*v*) were used as the vehicle control. All data are shown as the mean ± SEM of triplicate values. Statistical significance was analyzed by one-way ANOVA, Dunnett test. # *p* < 0.05 versus the glutamate (Glu.) control. (**b**) Qualitative analysis of intracellular ROS in glutamate-induced HT-22 cells observed under fluorescence and differential interference contrast (DIC) microscopes at 10× magnification. The scale bar indicates 10 µm.

At 5 mM, glutamate raised the intracellular reactive oxygen species (ROS) production by approximately 70% above the ROS level of the untreated HT-22 cells after 12 h of treatment. Under the same conditions with the addition of 0.5 mM of NAC, the reduction of intracellular ROS was at the level observed in the untreated HT-22 cells. The co-treatments of APE at 5, 10, 20, and 40 µg/mL or APH at 40 µg/mL with glutamate significantly reduced intracellular ROS generation in the HT-22 cells compared to glutamate control. Unfortunately, APW did not show the intracellular ROS inhibition on the glutamate-induced HT-22 cells (Figure 4a). Qualitative imaging of ROS accumulation in the HT-22 cells at the different conditions of treatments is presented in Figure 4b.

### 2.6. The Effect of APE Crude Extract on mRNA Expression of Antioxidant Enzymes in Glutamate-Induced HT-22 Cells

**Figure 5:**
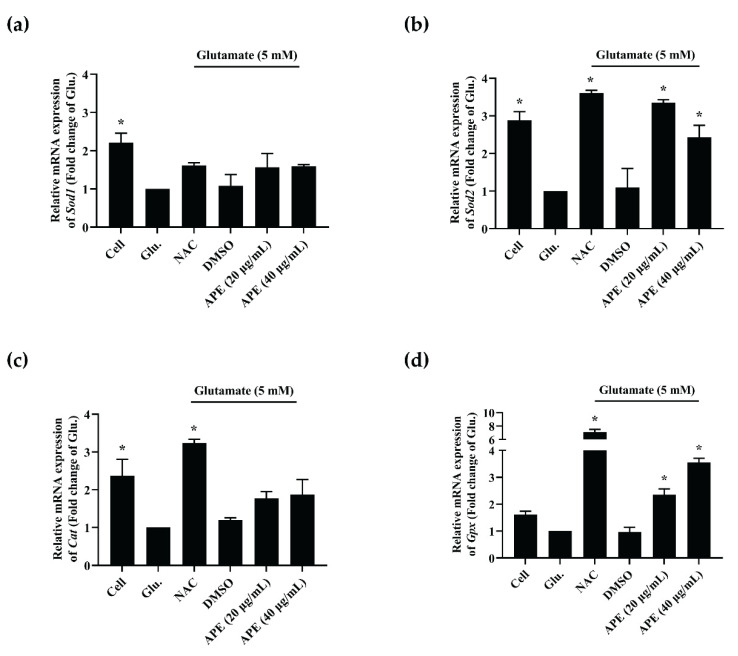
Antioxidant enzymes mRNA expressions: (**a**) *Sod1*, (**b**) *Sod2*, (**c**) *Cat*, and (**d**) *Gpx* genes of APE treatment on glutamate-induced HT-22 cell for 12 h. *N*-acetyl cysteine (NAC, 0.5 mM) and DMSO (0.1%, *v*/*v*) were used as the positive and vehicle control, respectively. All data are shown as the mean ± SEM of triplicate values. Statistical significance was analyzed by one-way ANOVA, Dunnett test. * *p* < 0.05 versus the glutamate (Glu.) control.

The APE extract exhibited the highest potential for neuroprotection and intracellular ROS inhibitory activity among all AP extracts. Thus, APE was further selected to study the effect on mRNA expression in antioxidant enzymes, namely Cu/Zn superoxide dismutase (*Sod1*), manganese superoxide dismutase (*Sod2*), catalase (*Cat*), and glutathione peroxidase (*Gpx*). These enzymes are responsible for cell protection against ROS-induced cellular damage [18]. The results showed that the treatment of 5 mM of glutamate downregulated the mRNA expression of these antioxidant enzyme genes compared to the untreated cells. In contrast, NAC could improve the levels of antioxidant enzyme gene expressions compared to glutamate control (Figure 5a–d). The co-treatment of APE and glutamate significantly increased the mRNA expressions of *Sod2* and *Gpx* genes compared to the glutamate treatment alone (Figure 5b,d). Unfortunately, the mRNA expressions of *Sod1* and *Cat* slightly increased. However, they did not show significant levels of gene upregulations (Figure 5a,c). These results indicate that the neuroprotective and intracellular ROS inhibition on glutamate-induced HT-22 cells of APE extract might be caused by the ability of the extract to upregulate antioxidant enzymes, particularly manganese superoxide dismutase and glutathione peroxidase [19].

### 2.7. The Effect of AP Crude Extracts on C. elegans Lifespan

**Figure 6:**
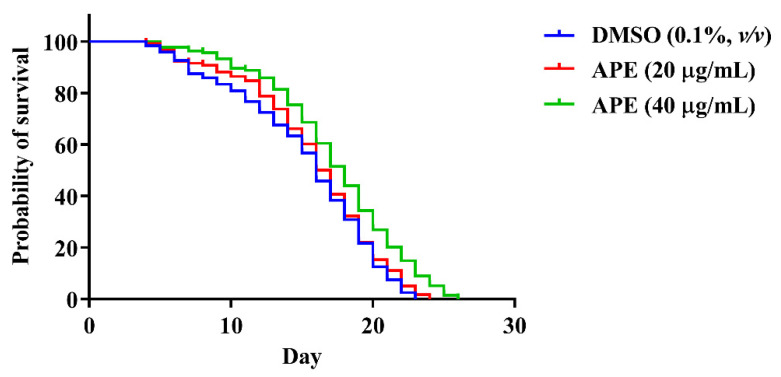
The effect of APE crude extracts on *C. elegans* lifespan.

Many studies have suggested that the lifespan could be prolonged by the antioxidant activity found in the diet [20,21,22,23]. According to our recent finding, APE exhibited antioxidant properties by scavenging free radicals, inhibiting intracellular ROS accumulation, and upregulating antioxidant enzymes. Thus, we further aimed to investigate the association between antioxidation and lifespan extension of APE extract. A lifespan assay of wild-type *C. elegans* was performed to evaluate the anti-aging property of APE extract. As shown in the Figure 6 and Table 2, 40 µg/mL of APE significantly extended the lifespan of the worms compared to the DMSO control. The worms’ mean lifespan in the presence of 40 µg/mL of APE was 17.66 ± 0.41 days, which was longer than the DMSO control (15.20 ± 0.46 days) by approximately 16.18%.

### 2.8. The Effects of the APE Extract on Age-Related Decline in Pharyngeal Pumping of C. elegans

**Figure 7:**
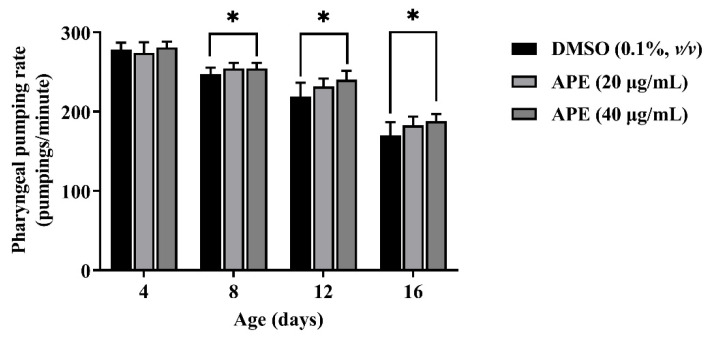
Effect of APE on age-related decline in pharyngeal pumping. It was observed that 40 µg/mL APE significantly attenuated the pharyngeal pumping rate in the *C. elegans* aging process. Statistical significance was analyzed by one-way ANOVA, Dunnett test. * *p* < 0.05 versus the DMSO of each time point.

To determine the effect of APE on *C. elegans* healthspan, the pharyngeal pumping rates were measured. Pharyngeal pumping is an age-related decline in muscle function and another marker for aging. At the L4 larvae stage, the nematodes were treated with APE (20 µg/mL and 40 µg/mL) and DMSO as the control. Then, the adult worms at the ages of 4, 8, 12, and 16 days were observed the pharyngeal pumping rate. The results showed that the pumping activity declined over time. Moreover, in the presence of 40 µg/mL of APE, the pharyngeal pumping rate increased significantly on days 8, 12, and 16 compared to the DMSO control group of each day (Figure 7). The results indicated that APE could improve the healthspan of aging worms.

### 2.9. The Effect of the APE Extract on C. elegans Spawning

**Figure 8:**
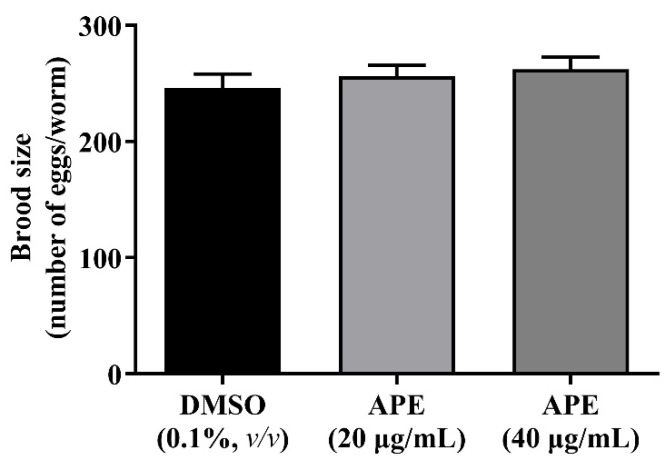
Effect of APE on *C. elegans* spawning. APE treatment did not affect the number of eggs laid by a worm versus the DMSO control group. Statistical significance was analyzed by one-way ANOVA (*p* < 0.05).

A brood size assay was used to determine the potential interfering effect of APE on the reproductive system of *C. elegans*. Total eggs and progenies of a single worm were measured after treatment with APE. There were no differences in brood size for APE-treated worms compared to the DMSO control group (Figure 8). The results indicated that APE treatment did not affect the nematode spawning.

### 2.10. Analysis of Lipophilic Compounds

**Figure 9:**
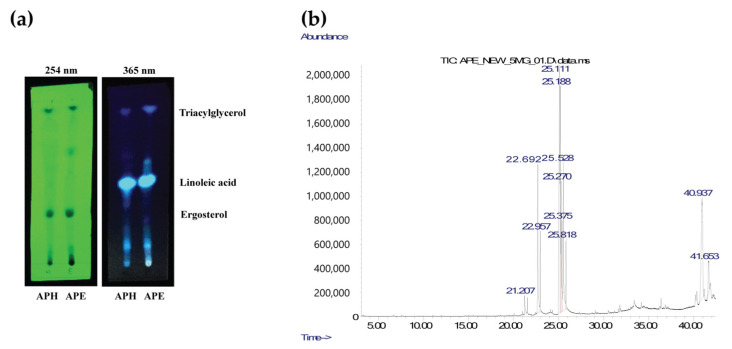
Analysis of lipophilic compounds in the AP crude extracts. (**a**) Thin-layer chromatography (TLC) of APH and APE was developed in hexane and ethyl acetate at a ratio of 8:2. The results were observed under UV light at 254 nm and 365 nm wavelengths. (**b**) Gas chromatogram of APE crude extracts analyzed by GC/MS.

The lipophilic compounds in APE crude extract were analyzed to identify the bioactive compound(s) that can prevent the glutamate toxicity and inhibit intracellular ROS. The APE crude extract was first analyzed by the TLC method, and the results were compared to that of APH crude extract. The chemical constituents of APH were thoroughly analyzed and reported in our previous study [4]. APH and APE crude extracts were applied onto a silica gel TLC plate, then developed in a hexane:ethyl acetate (8:2) mobile system. Finally, the spots of compound mobility were visualized under UV light at wavelengths of 254 and 365 nm. The TLC results showed that APH and APE extracts contain a similar composition of lipophilic compounds. These include triacylglycerol (linoleoyl, oleoyl, palmitoylglycerol), linoleic acid, and ergosterol with the retention values (R_f_) of 0.94, 0.49, and 0.30, respectively (Figure 9a). TLC analysis of APE extract estimated that linoleic acid is the major lipophilic compound in APE crude extract. GC/MS analysis was further performed to identify the essential oil presented in APE crude extract. The lipophilic chemical profile of APE crude extract is listed in Table 3. GM/MS results also confirmed that linoleic acid has the highest composition (262.3 mg/g lipophilic content in APE) among all identified compounds, followed by ergosterol (191.4 mg/g lipophilic content in APE).

### 2.11. Neuroprotective Effects of Linoleic Acid on Glutamate-Induced HT-22 Cell Toxicity

**Figure 10:**
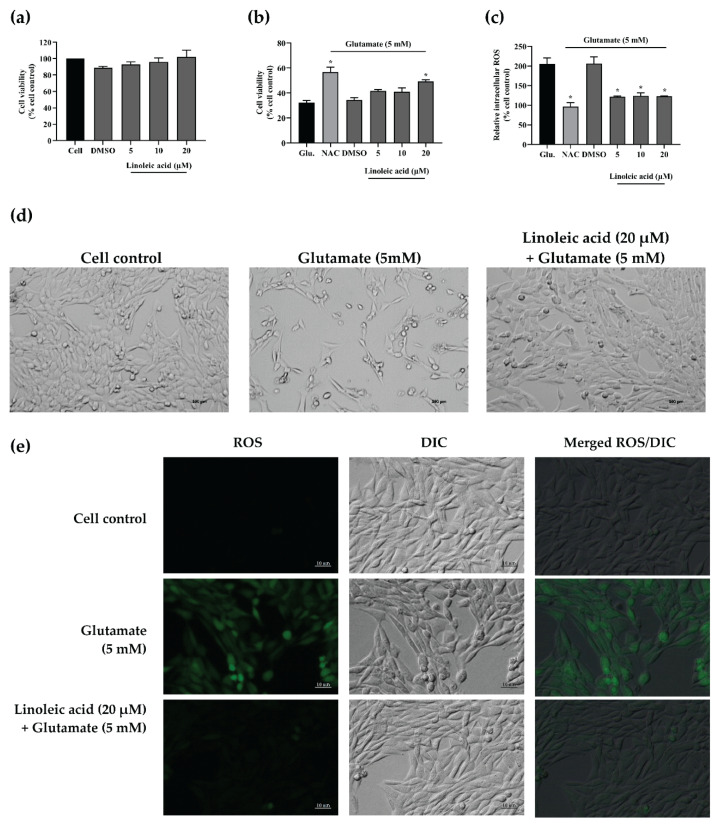
Neuroprotective effects of linoleic acid on glutamate-induced HT-22 cell toxicity. (**a**) Cytotoxicity of linoleic acid on HT-22 cells. (**b**) The effect of linoleic acid on glutamate-induced HT-22 cell death. (**c**) Quantitative analysis of intracellular ROS inhibitory activity of linoleic acid on glutamate-induced HT-22 toxicity. *N*-acetyl cysteine (NAC, 0.5 mM) and DMSO (0.1%, *v*/*v*) were used as the positive and vehicle control, respectively. All data are shown as the mean ± SEM of triplicate values. Statistical significance was analyzed by one-way ANOVA, Dunnett test. * *p* < 0.05 versus the glutamate (Glu.) control. (**d**) Morphological changes of glutamate-induced HT-22 hippocampal cells after co-treatment with linoleic acid (20 µM) for 12 h. (**e**) Qualitative analysis of intracellular ROS in glutamate-induced HT-22 cells observed under fluorescence and differential interference contrast (DIC) microscopes at 10× magnification. The scale bar indicates 10 µm.

Based on the high abundance of linoleic acid and the ability to cross the blood-brain barrier [24,25], linoleic acid was further investigated for its neuroprotective and antioxidant activities on glutamate-induced HT-22 neurotoxicity by evaluating the neuronal cell death and intracellular ROS accumulation. The cytotoxicity of linoleic acid on HT-22 cells was initially examined. The concentration of up to 20 µM of linoleic acid did not show noticeable toxicity in the HT-22 cells (Figure 10a). Moreover, linoleic acid at 20 µM significantly rescued the glutamate-induced toxicity in the HT-22 cells by increasing the cell viability by approximately 1.5-fold of the glutamate control (Figure 10b,d). The intracellular ROS investigation showed that linoleic acid (5 µM to 20 µM) significantly inhibited oxidative stress production in the glutamate-induced HT-22 cells (Figure 10c,e).

## 3. Discussion

Our current study investigated the therapeutic effects of AP crude extracts on oxidative stress inhibition, neuroprotection, lifespan extension, and healthspan improvement. We initially performed in vitro non-cell-based assays to evaluate the antioxidant activity of AP extracts. Our previous study identified the chemical constituents in APH. APH mainly comprised triacylglycerols, fatty acids, and terpenoids. No phenolics or flavonoids were identified in the chemical analysis of this extract [4]. This finding was supported by the weak antioxidant activity of APH (EC_50_ > 8 mg/mL). On the other hand, APW showed the highest total phenolic content among all the extracts. This was confirmed by evaluating the total phenolic content using the Folin-Ciocalteu method, which measures the water-soluble phenolic compounds [26]. The results correlate with the effective free radical scavenging activity (EC_50_ ≤ 3.62 mg/mL). Previous investigation of the antioxidant property in AP extracts showed that water extract provided higher antioxidant activity than the ethanol extract [27]. Chen Y et al. also found that polyphenols isolated from a hot-water extract of AP provided strong scavenging activities of the superoxide anion, hydroxyl radical, and DPPH radical [10]. This suggests that the water-soluble phenolic content of APW could offer an antioxidative effect. Furthermore, APE exhibited a high level of flavonoids and a moderate level of water-soluble phenolics with the EC_50_ of free radical scavenging at 3.11 mg/mL and 12.61 mg/mL for ABTS and DPPH assays, respectively. Our finding demonstrate that AP extracts contain antioxidant compounds that are flavonoids as well as water-soluble non-flavonoids phenolic compounds.

Although glutamate plays an essential role in neurotransmitters, a high glutamate level in the brain shows a risk of NDDs [16,28]. Several edible mushroom-derived products have been reported to attenuate glutamate-induced neurotoxicity [29,30,31]. Our study found that APE and APH could decrease the toxicity of glutamate on HT-22 cells. APE showed the most potential neuroprotection by suppressing intracellular ROS accumulation and neuronal death among all AP extracts. These results are supported by the antioxidant activity exhibited by APE in the in vitro non-cell-based ABTS and DPPH assays. In the case of APW, it showed more potent antioxidant activity than APE in the in vitro non-cell-based ABTS and DPPH assays. However, this extract showed no neuroprotection against glutamate-induced HT-22 cell damage. The lack of the activity may result from the poor cell permeability of the polar phenolic compounds.

Interestingly, the effect of AP extracts on ROS production was related to the effect on neuronal death. This finding indicates that the inhibition of ROS accumulation in the HT-22 cells may suppress glutamate-induced cell damage. Previous studies have shown that the ethanolic extracts of *Streblus asper*, *Aquilariae lignum*, and *Vitis vinifera* decreased intracellular ROS accumulation and protected against neuronal death in glutamate-induced HT-22 cells [18,32,33]. Ethanolic extract of tiger milk mushroom (*Lignosus rhinocerus*) presented high potential in neuroprotection against glutamate-induced cell damage, while cold and hot water extracts from this mushroom provided the unnoticeable neuroprotective effect [31].

The nuclear factor erythroid 2-related factor 2 (Nrf2) is a nuclear transcription factor that plays a crucial regulation of antioxidant-related genes such as NAD(P)H quinone dehydrogenase 1 (NQO1), glutamate-cysteine ligase modifier subunit (GCLM), and excitatory amino acid transporter 3 (EAAT3) [32]. Khan MM et al. found that the activation of Nrf2 signaling could suppress oxidative stress by increasing the mRNA expression of antioxidant enzymes, including GPx-1, CAT, and SOD1 [34,35]. Moreover, upregulation of Nrf2 expression was associated with oxidative stress inhibition and protection against neuronal death [36,37]. Many studies have demonstrated that natural products could attenuate neurotoxicity against glutamate via the Nrf2 pathway [19,32,36]. Our study found that APE could increase the mRNA expression of antioxidant enzymes. These studies have suggested that the protective effect of APE on glutamate-induced HT-22 neuronal death might be regulated via Nrf2 signaling by promoting antioxidant activity. However, the possible underlying mode of action of APE extract needs to be further investigated. Our findings and the literature indicate that natural products have influential roles in neuroprotection and the Nrf2 signaling pathway [19,32,36]. The investigation on the effects of the APE extract on the expression of Nrf2, Nrf2-related molecules, and the antioxidant-related genes are of importance. However, this investigation is outside the scope of this paper. This work will be carried in the future as part of our ongoing research.

Herein, we demonstrated that exposure to high-concentration of glutamate could exert antioxidant mechanisms by decreasing antioxidant enzymes and increasing oxidative stress, leading to neuronal damage [31,38]. In contrast, the treatment of NAC could attenuate the effects of glutamate. These findings agree with previous studies that NAC could activate antioxidant defense mechanisms via the Nrf2 pathway [39,40].

Moreover, APE crude extract dramatically increased the levels of antioxidant enzyme-related genes, including *Sod2* and *Gpx*, while the expressions of *Sod1* and *Cat* were slightly upregulated. These findings support that APE reduced ROS production in glutamate-induced HT-22 hippocampal cells. It has been reported that natural compounds isolated from plants and mushrooms, such as *Camellia sinensis*, *Anacardium occidentale* L., *Lignosus rhinocerus*, and *Glochidion zeylanicum*, could upregulate the mRNA expressions of antioxidant enzymes. In addition, these extracts could provide the cells with neuroprotection from glutamate-induced neuronal damage [31,36,41,42].

In vivo investigation of the *C. elegans* lifespan and healthspan effects, APE (40 µg/mL) improved the lifespan of *C. elegans* by prolonging the survival period. In addition, 40 µg/mL of APE promoted the healthspan of *C. elegans* by increasing the pharyngeal pumping rate, which is one of the age-related physical parameters [43,44]. Moreover, at this effective concentration, APE did not interfere with the reproductive system of the worms. These data reiterate the therapeutic anti-aging potential of AP. Hence, the underlying mechanisms of this effect are worthy of a throughout investigation. Previous studies have reported that natural products enhanced anti-aging activities through antioxidant, insulin/IGF-1, apoptotic, DAF-16/FOXO, and SIRT1/Nrf2 signaling pathways [36,45,46].

In vitro studies of APH and APE extracts showed that both extracts provided neuroprotective effects in glutamate-induced HT-22 cells. APH could protect the cells at high concentrations (≥40 µg/mL), while APE effectively protected the cells at lower concentrations (≥10 µg/mL). We hypothesized that the common compounds contained in APH and APE extracts might have neuroprotective activities. Thus, the lipophilic compounds of APE extract were identified in comparison to our reported chemical constituents in APH extract [4]. Although linoleic acid and ergosterol were detected in both APH and APE extracts, the ratios of these compounds in the extracts were different. APE extract presented a higher abundance of linoleic acid than that found in APH extract. Moreover, the TLC results demonstrated that both extracts were composed of a considerable amount of linoleic acid. Hence, the neuroprotective effects of pure linoleic acid were studied by evaluating its ability to protect against neuronal damage and intracellular ROS accumulation. We found that linoleic acid reduced glutamate-induced HT-22 cell toxicity and inhibited ROS accumulation in the cells. A previous study reported that linoleic acid could prevent age-dependent neuronal damage in a mouse model by hyperactivating Nrf2 signaling [47]. Moreover, many studies have indicated that supplements of polyunsaturated fatty acids, including linoleic acid, could ameliorate the progression of NDDs [47,48,49,50]. Chen XY et al. reported that coix (*Coix lacryma-jobi* L.) seed oil, which mainly contained linoleic acid, was responsible for prolonging the lifespan of *C. elegans* by reducing oxidative stress [51]. These studies suggest that linoleic acid may be a potential compound in APE extract responsible for antioxidant [52,53], neuroprotective [47,54,55], and anti-aging activities [51]. In addition, APE contained a higher abundance of flavonoids than APH, as shown in Figure 1b. We suggest that flavonoids may be other bioactive compounds that show antioxidant activity and provide a neuroprotective effect. Although in this study, we focused on lipophilic compounds, it would be interesting to identify the other compounds and further study their effects on antioxidant and neuroprotective activities.

Ergosterol was another major compound found in APE extract. Interestingly, ergosterol derivatives ganodermasides A, B, C, and D isolated from *Ganoderma lucidum* mushroom have been reported to have anti-aging activity [56,57]. Shao S et al. investigated ergosterol isolated from mushroom extracts for its antioxidant activity. They found that the composition of ergosterol showed a positive correlation with the antioxidative activity [58]. These data suggest that ergosterol might be another active compound which synergistically provides neuroprotective effects and presents anti-aging activity with linoleic acid.

In summary, the APE crude extract relieved hippocampal cell damage from glutamate-induced toxicity by upregulating *Sod1* and *Gpx* gene expressions and inhibiting intracellular ROS accumulation. In addition, APE extended the lifespan and healthspan of the *C. elegans* model. Chemical analysis of APE indicated that the extract was composed of high contents of fatty acids, ergosterol, and flavonoids. Our study is the first report of neuroprotective and lifespan/healthspan extension activities of AP extract. The findings serve as a novel pharmaceutical application of AP with benefits, particularly in NDDs and aging treatments.

## 4. Materials and Methods

### 4.1. Chemicals and Reagents

L-glutamic acid, Folin-Ciocalteu phenol reagent, sodium carbonate, gallic acid, aluminum chloride, sodium acetate, quercetin, 2,2′-Azino-bis (3-ethylbenzothiazoline-6-sulfonic acid) diammonium salt (ABTS), 2,2-Diphenyl-1-picrylhydrazyl (DPPH), *N*-Acetyl cysteine, and ascorbic acid were purchased from Sigma-Aldrich (St. Louis, MO, USA). Dimethyl sulfoxide (DMSO) was purchased from RCI Labscan (Bangkok, Thailand). Dulbecco’s Modified Eagle Medium (DMEM), fetal bovine serum (FBS), phosphate-buffered saline (PBS), and Hanks’ Balanced Salt solution (HBSS) were purchased from Thermo Scientific HyClone (Logan, UT, USA). Trypsin-EDTA (0.25%) was purchased from Gibco (Waltham, MA, USA). 3-(4,5-dimetylthiazol-2-yl)-2,5-diphenyltetrazoliumbromide (MTT) was purchased from Bio Basic (Markham, ON, Canada). Trizol was purchased from Invitrogen (Carlsbad, CA, USA). The maxime RT PreMix Oligo 18 Primer kit and RealMOD^TM^ Green W2 2x qPCR mix were purchased from iNtRON Biotechnology (Gyeonggi, South Korea). 2′, 7′-dichlorodihydrofluorescein diacetate (H_2_DCFDA) was purchased from Molecular Probes (Eugene, OR, USA). The annexin V-Fluorescein isothiocyanate (FITC) apoptosis detection kit was purchased from BioLegend (San Diego, IL, USA).

### 4.2. Crude Extract Preparation

All extracts were obtained from the same batch as our previous study [4]. Briefly, *Auricularia polytricha* (AP) dried fruiting bodies were sequentially macerated in hexane, ethanol, and water to obtain AP hexane (APH), ethanol (APE), and water (APW) extracts, respectively. Aliquots of each extract were obtained for chemical analysis. APH and APE were dissolved in absolute DMSO to produce the stock solution at 100 mg/mL, while APW was prepared in PBS at the same concentration. Each stock solution was further divided into workable aliquots. These aliquots were stored at −20 °C until they were needed for biological assays.

### 4.3. Total Phenolic Content Analysis

The total phenolic content of the AP extracts was determined by the Folin-Ciocalteu method [32]. Briefly, the extract at 1 mg/mL (50 µL) was mixed with the Folin-Ciocalteu’s phenol reagent (10%, *v*/*v*; 50 µL) in a 96-well plate, and then the mixture was incubated in the dark at room temperature for 20 min. Next, 7.5% (*w*/*v*) of sodium carbonate (Na_2_CO_3_, 50 µL) was added and further incubated for 20 min. The absorbance at 760 nm was measured using a microplate reader. Gallic acid was used as a standard compound, the various concentrations of gallic acid were tested to generate a standard calibration curve. The extract’s total phenolic content was calculated using the calibration curve and reported in mg of gallic acid equivalent (GAE)/g dry weight.

### 4.4. Total Flavonoid Content Analysis

Total flavonoid content was examined by the colorimetric aluminum chloride method [32]. Briefly, 1 mg/mL (50 µL) of the extract was mixed with 10% (*v*/*v*) aluminum chloride (AlCl_3_, 10 µL), 1 M sodium acetate (NaOAc, 10 µL), and 95% (*v*/*v*) ethanol (150 µL). The mixture was incubated in the dark at room temperature for 40 min. Then, the absorbance at 415 nm was measured using a microplate reader. The total flavonoid content of the extracts was calculated by comparing it to the quercetin standard calibration curve. The result was reported in mg of quercetin equivalent (QE)/g dry weight.

### 4.5. Free Radical Scavenging Assays

#### 4.5.1. ABTS Assay

The ABTS solution was prepared by mixing 7 mM of ABTS and 2.45 mM potassium persulfate (K_2_S_2_O_8_). The solution was incubated overnight to generate the stable free radical cation (ABTS^•+^). Then, 20 µL of AP crude extracts (0, 0.5, 1, 2, 4, and 8 mg/mL) was added to 180 µL of ABTS solution and placed in the dark for 20 min. The ABTS^•+^ was decolorized from green to colorless as the reaction continued, and a microplate reader measured the absorbance at the wavelength of 734 nm.

#### 4.5.2. DPPH Assay

DPPH (the stable free radical (DPPH^•^)) was prepared at a 0.2 mM solution in ethanol. After adding 20 µL of the sample to 180 µL of the DPPH solution for 20 min in the dark, the purple DPPH solution was neutralized to yellow. The color change in the reaction was read at the wavelength of 517 nm.

The antioxidant capacity of AP crude extracts was calculated by comparing it to the vitamin C calibration curve. The results were expressed as mg of vitamin C equivalent antioxidant capacity (VCEAC)/g dry weight. Various concentrations of AP crude extracts were tested to determine the half-maximal effective concentration (EC_50_) value.

### 4.6. Cell Culture

The HT-22 mouse hippocampal cell line was obtained from Professor David Schubert, Salk Institute, San Diego, CA, USA. The cells were maintained in DMEM supplemented with 10% FBS and grown in a humidified incubator with 5% CO_2_ at 37 °C.

### 4.7. HT-22 Cytotoxicity of Crude Extracts

HT-22 cells (5 × 10^3^ cells/well) were cultured on a 96-well plate overnight then treated with crude extracts or compounds at designed concentrations for 24 h. An MTT assay was performed to determine the cytotoxicity of extracts and compounds following the manufacturer’s instructions. MTT (20 µL) was added to each well of 96-well plate that contained 200 µL of cell culture media. Then, the cell media and MTT were incubated for 3 h. After that, the culture media and MTT were removed and replaced with absolute DMSO (200 µL) to dissolve formazan crystal as a final product of the MTT assay [59,60]. The absorbance at 570 nm was measured using a microplate reader. The percentage of cell viability was calculated by compared to the cell control, an untreated cell.

### 4.8. Glutamate-Induced HT-22 Neurotoxicity

HT-22 cells (5 × 10^3^ cells/well) were plated and incubated overnight. Glutamate was freshly prepared in 10% FBS-supplemented culture media. The cells were treated with 5 mM of glutamate combined with crude extracts or compounds for 12 h. The treatment of glutamate alone was used as a glutamate control (Glu.). The cotreatment of glutamate and DMSO (0.1%, *v*/*v*) or PBS (0.1%, *v*/*v*) was utilized as the vehicle control of the AP extracts. The HT-22 cell viability was measured by an MTT assay. The morphological changes of the HT-22 cells after treatments were observed under a differential interference contrast (DIC) microscope.

### 4.9. Intracellular Reactive Oxygen Species (ROS) Assay

HT-22 cells (2 × 10^4^ cells/well) were seeded on a 96-well black plate with a clear bottom and cultured overnight. The cultured cells were cotreated with glutamate (5 mM) and AP crude extracts or NAC for 12 h. After treatments, the cells were washed twice with warm HBSS. Then, the cells were incubated with H_2_DCFDA (10 µM, diluted in serum-free culture media) for 30 min. The fluorescence (excitation/emission = 485/535 nm) was measured using an EnSpire Plate Reader (Perkin-Elmer). For qualitative analysis, HT-22 cells were plated on a 35-mm dish at a cell density of 6 × 10^5^ cells overnight. Then, the cells were treated and stained with H_2_DCFDA following the previous procedures. The intracellular ROS and the cell confluence of the stained cells were observed under fluorescence and DIC microscopes, respectively.

### 4.10. RNA Isolation and Reverse Transcription-Quantitative Real-Time Polymerase Chain Reaction (RT-qPCR)

HT-22 cells were treated with the designed treatments as described above. After that, total RNA was isolated using Trizol reagent (Invitrogen) according to the manufacturer’s instructions. The amount of the isolated RNA was measured with an absorbance at 260 nm using a NanoDrop spectrophotometer. Then, 1 µg of the isolated RNA was converted to complementary DNA (cDNA) using Maxime RT PreMix Oligo 18 Primer kit (iNtRON Biotechnology). The cDNA templates were amplified using RealMOD™ Green W2 2x qPCR mix (iNtRON Biotechnology) with the specific primers of SOD1, SOD2, CAT, GPx, and β-actin, following the previous study of Sukprasansap M et al. [19]. All PCRs were performed using an Exicycler^™^ 96 (Bioneer). Quantitative gene expression was calculated by the 2^−ΔΔCT^ method with β-actin normalization.

### 4.11. Caenorhabditis elegans Maintenance, Synchronization, and Treatment

In this study, the wild-type N2 Bristol strain of *C. elegans* was utilized. The worms were maintained on a nematode growth medium (NGM) supplemented with *E. coli* OP50 in an incubator at 20 °C. The nematodes and *E. coli* OP50 were obtained from Caenorhabditis Genetics Center (CGC), University of Minnesota, USA.

The isolation of eggs from gravid hermaphrodites was performed to synchronize the stage of the nematodes. Briefly, the lysis solution (5 M of NaOH and 5% NaOCl) was added to lyse the gravid hermaphrodites for 10 min under the vortex condition. The solution was centrifuged at 2000 rpm for 2 min to collect a pellet containing eggs. Then, the eggs were washed twice with M9 buffer and resuspended in the same buffer. The collected eggs were hatched overnight in a 20 °C incubator to obtain the L1 larvae. The L1 larvae were further cultured on NGM containing *E. coli* OP50 to synchronize at the L4 larvae stage for 2 days.

For the treatment of *C. elegans*, the nematodes were separated into three groups, including a vehicle control group (0.1%, *v*/*v* of DMSO) and two extract treatment groups: APE at 20 µg/mL and 40 µg/mL. All treatments were prepared in S-medium containing *E. coli* OP50 (OD_600_ = 1.0) and spread on NGM agar plates.

### 4.12. Lifespan Assay

The L4 larvae nematodes were cultured on NGM agar supplemented with the designed treatments in an incubator at 20 °C. The worms were moved to the fresh treatment agar every day to exclude the interferences from starvation and the new population. The numbers of live and dead worms were recorded every day until all the worms had died. The worms that showed no response to stimulus and no pharyngeal pumping movement were considered as dead.

### 4.13. Pharyngeal Pumping Rate

A pharyngeal pumping rate assay was performed to determine the age-related decline in muscle function. At the L4 stage, the synchronized nematodes were grown on NGM containing *E. coli* OP50 and treatments. During the reproductive period, the adult nematodes were transferred daily to the fresh treatment agar. The pharyngeal pumping rate of the adult worms was counted per minute after 4, 8, 12, and 16 days of treatment.

### 4.14. Brood Size Assay

The toxicity of APE extract against *C. elegans* was determined using the brood size assay. The assay was performed by evaluating the fertility rate of the worms. A single L4-stage nematode was maintained on NGM agar supplemented with *E. coli* OP50 and treatments at 20 °C. The adult worms were transferred to the fresh agar every day. The number of eggs laid by each worm was observed daily and counted until the termination of egg-laying.

### 4.15. Statistical Analysis

All experiments were performed on three independent replicates and reported as the mean of the three experiments ± standard error of the mean (SEM). The data were statistically analyzed by one-way ANOVA, followed by the Dunnett post hoc test using GraphPad Prism 9 software. For lifespan analysis, the treatment group was compared to the DMSO control by log-rank (Mantel-Cox) tests followed by the Gehan−Breslow−Wilcoxon test. The *p*-values less than 0.05 were considered statistically significant.

### 4.16. Thin-Layer Chromatography (TLC)

Qualitative analysis of the chemical constituents of the APE crude extract was performed by thin-layer chromatography (TLC). The extract was spotted on the silica TLC plate (silica gel 60 F254 on aluminum sheet). Then, the plate was placed in a jar containing hexane:ethyl acetate (8:2) as a mobile phase system. The results were observed under ultraviolet light at wavelengths of 254 nm and 365 nm. The retention factor (R_f_) values of each isolated compound were calculated and compared to the R_f_ values of known compounds.

### 4.17. Gas Chromatography/Mass Spectrometry (GC/MS)

Gas chromatography/mass spectrometry (GC/MS) analysis of lipophilic constituents of APE was performed using Agilent 19091P-M15 with column HP-PLOT AI203 (50.0 m × 320 µm × 8.00 µm). The sample was analyzed using a split ratio of 15:1, 1.5 mL/min of He flowrate with an oven temperature of 50 °C to 220 °C (2 min) at 7 °C/min, then 270 °C (5 min) at 7 °C/min, followed by 290 °C at 5 °C/min. The molecular masses were searched using the NIST MS search 2.0 Data analysis software. The number of identified compounds was calculated compared to total lipophilic compounds in APE extract and reported as mg/g lipophilic content in APE.

## 5. Conclusions

The current study revealed the effects of AP extracts on oxidative stress inhibition and neuroprotection in glutamate-induced neuronal toxicity. Among all the extracts, APE exhibited the most potent effects on the inhibition of intracellular ROS accumulation and protection against neuronal death in glutamate-induced HT-22 hippocampal cells. Moreover, APE could attenuate the expressions of antioxidant-related genes, including *Sod2* and *Gpx*. In vivo investigations showed that APE promoted the longevity and improved the healthspan of the wild-type *C. elegans* model. Chemical analysis of APE indicated that the extract is mainly composed of linoleic acid, ergosterol, and flavonoids. These findings suggest that APE is an exciting source of natural therapeutic compounds that potentially serve as neuroprotectant and anti-aging agents.

## Figures and Tables

**Table 1:** Antioxidant capacity and half-maximal effective concentration (EC_50_) of free radical scavenging activity of AP crude extracts.

Crude Extract	ABTS Scavenging Activity		DPPH Scavenging Activity	
	mg VCEAC/g Dry Weight	EC_50_ (mg/mL)	mg VCEAC/g Dry Weight	EC_50_ (mg/mL)
APH	0.92 ± 0.12 ^a^	>8	0.99 ± 0.08 ^a, b^	>8
APE	1.57 ± 0.04	3.11 ± 0.05	1.27 ± 0.04 ^a, c^	12.61 ± 3.47
APW	2.47 ± 0.06 ^a^	1.06 ± 0.04	1.88 ± 0.25 ^b, c^	3.62 ± 0.35

The data are expressed as mean ± SEM (*n* = 3). The same superscript letters in the same column indicate a significant difference between the means by one-way ANOVA (*p* < 0.05).

**Table 2:** Results and statistical analyses of the lifespan of *C. elegans* treated with APE.

Treatment	Mean Lifespan		Maximum Lifespan		*p*-Value (vs. Control)	Number of Worms
	Day ± SEM	% Increase (vs. Control)	Day ± SEM	% Increase (vs. Control)		
DMSO (0.1%, *v*/*v*)	15.20 ± 0.46	-	22.60 ± 0.24	-	-	*N* = 120
20 µg/mL APE	15.86 ± 0.43	4.34%	23.50 ± 0.20	3.98%	0.4017	*N* = 118
40 µg/mL APE	17.66 ± 0.41	16.18%	25.33 ± 0.21	12.08%	0.0013 *	*N* = 131

* Indicates statistical significance vs. DMSO control analyzed using log-rank (Mantel-Cox) tests followed by the Gehan−Breslow−Wilcoxon test, *p* < 0.05.

**Table 3:** Lipophilic compounds of APE crude extract analyzed by GC/MS.

RT (min)	Identified Compound	Amount (mg/g Lipophilic Content in APE)
21.206	Pentadecanoic acid	14.7
22.693	Palmitic acid	105.5
22.958	Palmitic acid, ethyl ester	28.7
25.111	Linoleic acid	262.3
25.187	*cis*-Vaccenic acid	116.7
25.271	Linoleic acid, ethyl ester	69.3
25.375	Oleic acid, ethyl ester	46.3
25.527	Stearic acid	98.4
25.817	Stearic acid, ethyl ester	32.4
40.936	Ergosterol	191.4

## Data Availability

Data is contained within the article.
